# Notes and News

**Published:** 1896-08-29

**Authors:** 


### Notes and News.

(Collected and Communicated.)

The monument to the memory of Alphonse Guerin, erected by the Bretons of Paris, will be unveiled at Marbihan on September 14th.

The Hackney pauper schools are remarkable in propagating disease. Out of 300 children housed 126 are in the infirmary at the present time.

It is felt very necessary that Melbourne should be provided with an infectious diseases hospital. The general hospitals do not receive fever cases, and the managers of the Alfred Hospital refused an application for the use of an empty wing for the purpose. Public opinion is aroused, but nothing definite has yet been accomplished.

An extraordinary trial was held recently at Magdeburg, in Germany, in which the State prosecuted. The accusation was against a doctor for the non-administration of alcohol to a patient, death being attributed to this cause. The case was lost, the General Medical Council of Saxony giving opinion that it was inadvisable to put any limit to the exercise of the individual judgment of the physician on the subject of alcohol.

A matter of considerable importance is just now exercising the committees of the various hospitals in Sydney, and that is the fact that the Water and Sewerage Board intend to sue for rates. Water has never been charged for, although the consumption is checked by meter; but the sewerage-rate papers have been delivered for some years. The Sydney Hospital paid up for a few years, but, finding that the Prince Alfred Committee were shelving their papers, they did likewise. Now the Water and Sewerage Board's solicitor has served the Prince Alfred Hospital Committee with a notice that, unless payment is made by a certain date, they will be sued for the whole amount, which has reached the respectable total of £780. This will mean a serious thing for this hospital should it be compelled to pay. A Bill for the regulation of the Water and Sewerage Board is now before the Legislative Assembly, and it is hoped that a clause to exempt the hospitals from sewerage as well as water rates may be included and agreed to.

No less than 16,000 people are reported to have been rendered ill from the recent great heat in New York. As many as 288 deaths were reported in eight days as directly due to this cause.

Plans for providing Coventry with new fever and small-pox hospitals are being considered. If the present fever hospital be extended an expenditure of £6,700 is regarded as necessary, and for a new small-pox hospital £31,000.

The system of subsidising is one that does not apparently commend itself to the English mind. Nevertheless, a little encouragement in some directions may be the means of attaining great benefits to the majority. If, as it is contended, the use of gas for cooking has already effected an improvement in the atmosphere of our metropolis, why should not the municipality do something for the good of the whole community by subsidising the erection of gas stoves in the poorer dwellings, for there can be little doubt that the small domestic fireplaces are the chief cause of the smoky air of towns.

There is a naïve directness in the Government reports on hospitals and kindred institutions in our Colonies which is almost startling. For instance, in the report of the medical officers at the Queenstown Hospital, Cape of Good Hope, we read that though it is interesting to receive cases of typhoid and dysentery into the wards, the absence of a system of sewers and of a destructor furnace renders the sanitary disposal a source of anxiety, and exercises the ingenuity of the committee of management not a little.

The work of the Italian Red Cross Society is reported as most excellent. During the late war in Africa nearly 4,000 patients were ministered to. The staff sent out consisted of fourteen surgeons, two dispensers, three head inspectors, nine inspectors, forty-eight dressers, and sixteen servants. The society established a hospital at Naples to receive patients on their return to Italy, the Duchess of Ravaschieri opening a hospital for convalescents at the same place. Not only did the society tend the wounded, but it provided money for those patients who needed it on leaving the hospitals. A sum of £5[illegible],426 was subscribed in Italy and abroad for the succour of the wounded, all of which was not expended. The founder of the Red Cross Society, now so important an organisation throughout Europe, is reported to be living in poverty. The Mayor of Stuttgart is making a collection on his behalf.

In an article in *Science Progress* by Professor Halliburton, of King's College, attention is drawn to the recent discovery of iodine as a constituent of the animal organism. In the course of researches which were being made with the object of discovering the active chemical substance secreted by the thyroid gland, E. Baumann came across a substance which he called thyro-iodin, which is remarkable among animal products in containing iodine. Almost simultaneously with Baumann's announcement it was shown by Drecksel that iodine occurred in quite a different part of the animal kingdom, viz., in the horny skeleton of the gorgonia. In view of the fact that as an integral part of living animal structures iodine has hitherto not been known to exist, its demonstration is of great importance, and is one of the most startling of scientific discoveries made of recent years in the domain of chemical physiology.

